# Virtual arthroplasty follow-up (VARF) in total hip replacement: a safe, effective, and sustainable model for post-operative care

**DOI:** 10.1007/s00590-025-04479-y

**Published:** 2025-08-26

**Authors:** Nicholas A Platt, Benjamin Bolland

**Affiliations:** 1 University of Bristol, Bristol, UK; 2 Musgrove Park Hospital, Taunton, UK

**Keywords:** Arthroplasty, Follow-up, Total hip replacement, Total hip arthroplasty, Sustainability, Net zero

## Abstract

**Purpose:**

Virtual follow-up programmes offer an alternative to traditional in-person appointments, aiming to reduce patient burden and optimise healthcare resources. Following a successful pilot in 2020, the virtual arthroplasty follow-up (VARF) pathway was formally implemented to monitor total hip replacement (THR) patients. This study evaluates the clinical outcomes, patient satisfaction, economic impact, and environmental benefits of VARF in a large cohort.

**Methods:**

A retrospective analysis was performed on a prospectively maintained database of 319 patients who underwent primary THR between January 2022 and July 2023. Follow-up was conducted using Oxford Hip Scores (OHS) and radiographs. Patient satisfaction was assessed via telephone survey in a representative sample of 35 patients. Estimated carbon dioxide emissions related to clinic visits were calculated based on previously published data.

**Results:**

A total of 92.2% (*n* = 294) of patients were successfully managed through VARF. Patient satisfaction was high, with 86% of respondents reporting they were ‘satisfied’ or ‘very satisfied’, and 69% perceiving time and cost-savings. Financial analysis demonstrated an average saving of £11.78 per patient in fuel and parking costs. A theoretical cost reduction of £48,978.91 was identified for the Trust, primarily due to operational efficiencies and the release of clinic time. While not a direct monetary saving, this freed capacity allowed reallocation for other clinical purposes, enhancing overall service efficiency. Environmentally, the programme prevented an estimated 19,691 kg of CO_2_ equivalent emissions by reducing in-person visits.

**Conclusion:**

VARF is a safe, effective, and patient-centred approach for THR follow-up, achieving high satisfaction rates while delivering significant cost and environmental benefits. This model demonstrates strong potential for wider adoption in arthroplasty post-operative care.

**Supplementary Information:**

The online version contains supplementary material available at 10.1007/s00590-025-04479-y.

## Introduction

In recent years, the healthcare landscape in the UK has undergone a significant shift towards digital and virtual care models, particularly within orthopaedics [1, 2]. Among these innovations, virtual follow-up programmes for arthroplasty patients have emerged to improve post-operative care while optimising resource allocation [3, 4]. Traditionally, arthroplasty follow-up has relied on in-person appointments, requiring patients to travel for routine assessments, imaging, and consultations [5].

Virtual follow-up offers enhanced convenience for patients, reduces the logistical burden on healthcare services, and supports the NHS commitment to achieving ‘net zero’ emissions [6–8].

A virtual arthroplasty follow-up (VARF) programme for hip arthroplasty patients was piloted at Musgrove Park Hospital (MPH), a large district general hospital in Taunton, Somerset, between 2020 and 2021. Prior to this, standard follow-up included face-to-face appointments at 6 weeks, 1 year, 5 years, and 10 years post-operatively, with an additional 7 year review for patients under 50 at the time of surgery.

Under VARF, the initial 6 week review was conducted via video consultation. At 1 year, patients were invited to attend local imaging facilities for radiographs and complete patient-reported outcome measures (PROMs), including the Oxford Hip Score (OHS) and University of California, Los Angeles (UCLA) activity score [9, 10]. Clinicians reviewed these data to assess recovery and guide further management remotely.

The pilot study, published in 2022, demonstrated VARF to be a clinically safe and effective approach [11]. It showed high satisfaction (90%), excellent functional outcomes (79% with good or very good OHS), and a low rate of conversion to in-person appointments (4%). Additionally, it provided significant time and cost-savings for both patients and the Trust, while reducing environmental impact.

Building on these promising results, VARF was adopted as the standard follow-up pathway for hip arthroplasty patients at MPH. The pilot study was conducted during a period of reduced elective capacity in the aftermath of the COVID-19 pandemic. This study therefore aims to evaluate the performance and impact of VARF following its full implementation, at a time when elective activity had returned to normal levels.

## Methods

**Fig. 1:**
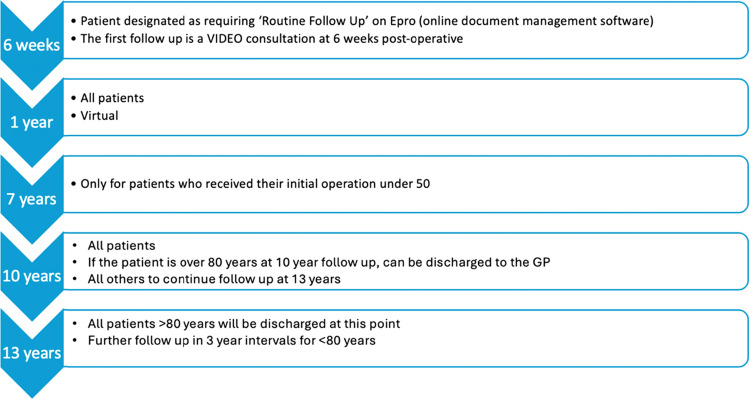
Review algorithm

A retrospective case series was conducted on 379 patients who underwent primary THR at Musgrove Park Hospital between January 2022 and July 2023. The review algorithm, outlined in Fig. 1, followed the same structure as the previously published pilot study and remained unchanged throughout this evaluation.

Following a wound review at their GP practice 2 weeks post-operatively, patients attended a 6 week video consultation with a consultant via Microsoft Teams. A modified virtual clinical assessment was conducted, including inspection of the surgical wound, assessment of gait, and evaluation of range of motion (ROM). Those deemed suitable for ongoing virtual follow-up were sent an information pack ahead of their 1 year review. This included instructions for completing patient-reported outcome measures (PROMs), guidance on arranging a radiograph at a local NHS treatment centre, and a prepaid envelope for returning completed forms.

The PROMs included the Oxford Hip Score (OHS) and the University of California, Los Angeles (UCLA) Activity Score. Radiographs were obtained locally for patient convenience and uploaded to the hospital PACS system. All images were reviewed by a consultant hip surgeon to assess implant positioning, loosening, and evidence of osteolysis. Image quality was confirmed prior to final interpretation.

Once both PROMs and radiographs were returned and deemed satisfactory, patients were booked into the virtual arthroplasty follow-up (VARF). If either the OHS or radiograph was considered suboptimal, the patient was contacted by the Arthroplasty Clinical Nurse Practitioner (CNP) for further clinical triage. Based on this assessment, patients were either:

(i) Re-enrolled into the VARF pathway,
(ii) Reviewed via telephone consultation with a consultant, or
(iii) Referred for face-to-face clinical assessment with a consultant.

**Fig. 2:**
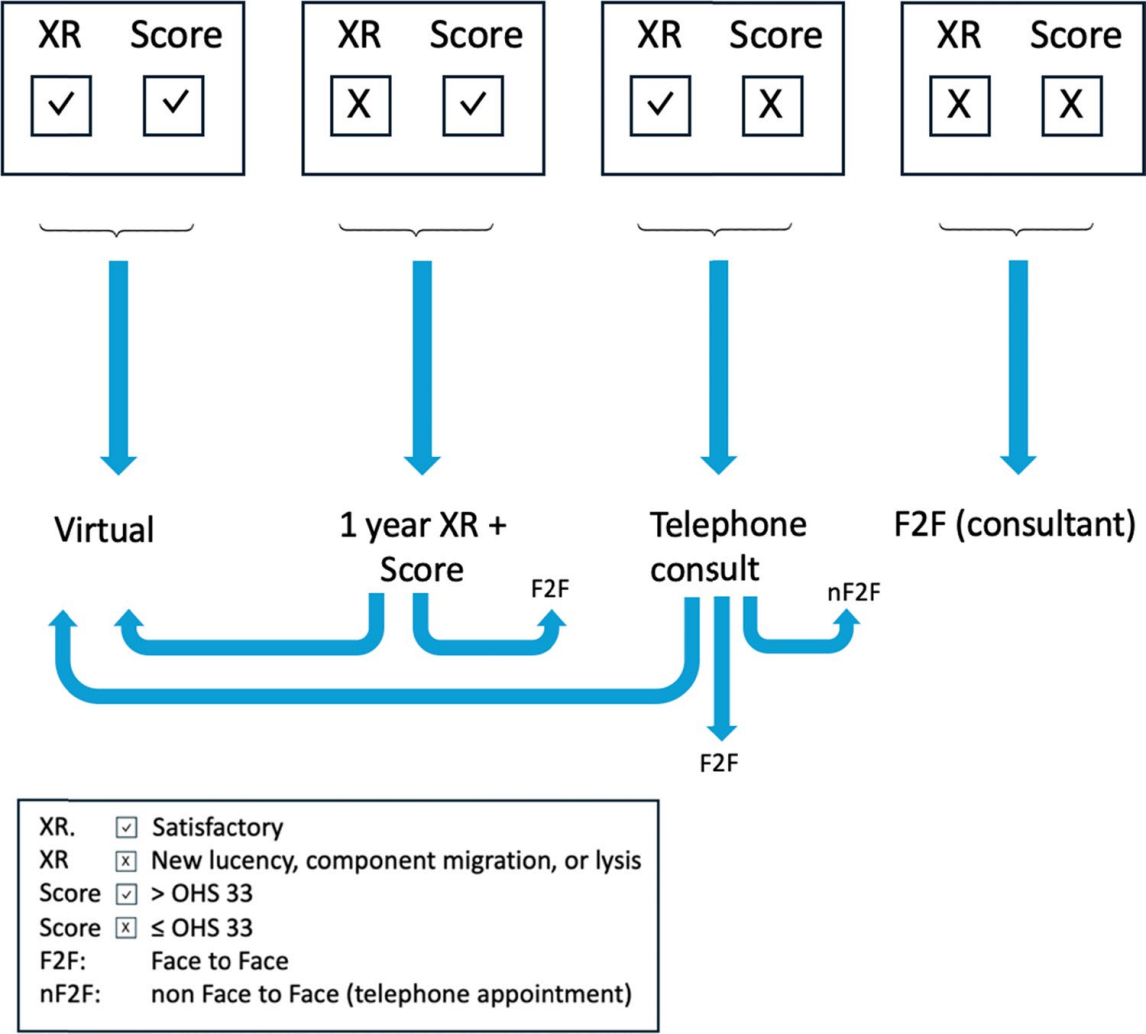
Triage protocol at 1 year virtual follow-up

This triage process is illustrated in Fig. 2.

Patients with both an acceptable radiograph and PROM score were booked directly into the VARF pathway, with an OHS greater than 33 considered acceptable. Radiographs were deemed unsuitable for automatic VARF enrolment if consultant review identified new lucency, component migration, or lysis. In such cases, a consultant determined whether the patient should be seen face-to-face or re-enrolled into the VARF pathway.

Patients with an OHS 33 or less were contacted by a Clinical Nurse Practitioner (CNP) for further triage. Based on this telephone assessment by a CNP, they were either referred for face-to-face consultation (F2F) with a consultant, a non-face-to-face (nf2f)/telephone consultation with a consultant, or re-enrolled into the VARF pathway. These cases were all discussed with the consultant to determine appropriate follow-up following summary of telephone consultation of CNP with patient.

Patients who remained well at 1 year review with satisfactory radiographs and OHS were retained in the VARF pathway. In cases requiring face-to-face or consultant telephone assessment, patients were temporarily removed from VARF but, if deemed stable, were re-enrolled for follow-up according to the VARF protocol.

Descriptive statistics were used to summarise patient characteristics, outcomes, and satisfaction scores. Continuous variables are presented as means or medians with ranges, and categorical data as counts and percentages. No inferential statistics were applied due to the descriptive nature of this case series.

To evaluate patient satisfaction, a random sample of 35 patients was contacted by telephone. A structured questionnaire (Appendix A) included multiple-choice questions and open-ended responses assessed using a Likert scale.

Cost analyses were informed by data from the original pilot study, with financial figures supplied by the hospital management team. Environmental impact was evaluated using previously published carbon footprint data, with emissions reported in terms of carbon dioxide equivalent (CO_2_e). CO_2_e provides a more comprehensive measure than CO_2_ alone, as it accounts for all greenhouse gases expressed in terms of their CO_2_ impact. Estimates of CO_2_e emissions from hospital lighting, heating, and waste generation were included in the assessment.

Following the 1 year virtual review, patients who remain clinically well with satisfactory radiographs and PROMs will continue within the virtual arthroplasty follow-up (VARF) pathway. As per the established protocol, patients will only exit VARF if a clinical concern arises that necessitates telephone or face-to-face review with a consultant. Importantly, if this review confirms no further issues, patients can be re-integrated into the VARF schedule, continuing their follow-up virtually at the defined time points. This approach ensures safe, efficient, and sustainable long-term monitoring while maintaining flexibility for clinical escalation when needed.

## Results

Of the initial 379 patients identified, 60 were excluded from the final analysis. This included five patients who had died, 11 who had relocated out of area, three who opted for private follow-up, and one patient who declined further follow-up, reporting satisfaction with their outcome. The remaining 40 patients were classified as did not attend (DNA). Of these, six did not attend for radiographs, three failed to return their questionnaires, and four were uncontactable by telephone. The remaining 27 were recorded as DNA with no additional information, having failed to attend for radiographs despite two reminder letters and no response to a follow-up phone call. This left a total of 319 patients for inclusion in the final analysis.

The following data represent outcomes at the 1 year follow-up.
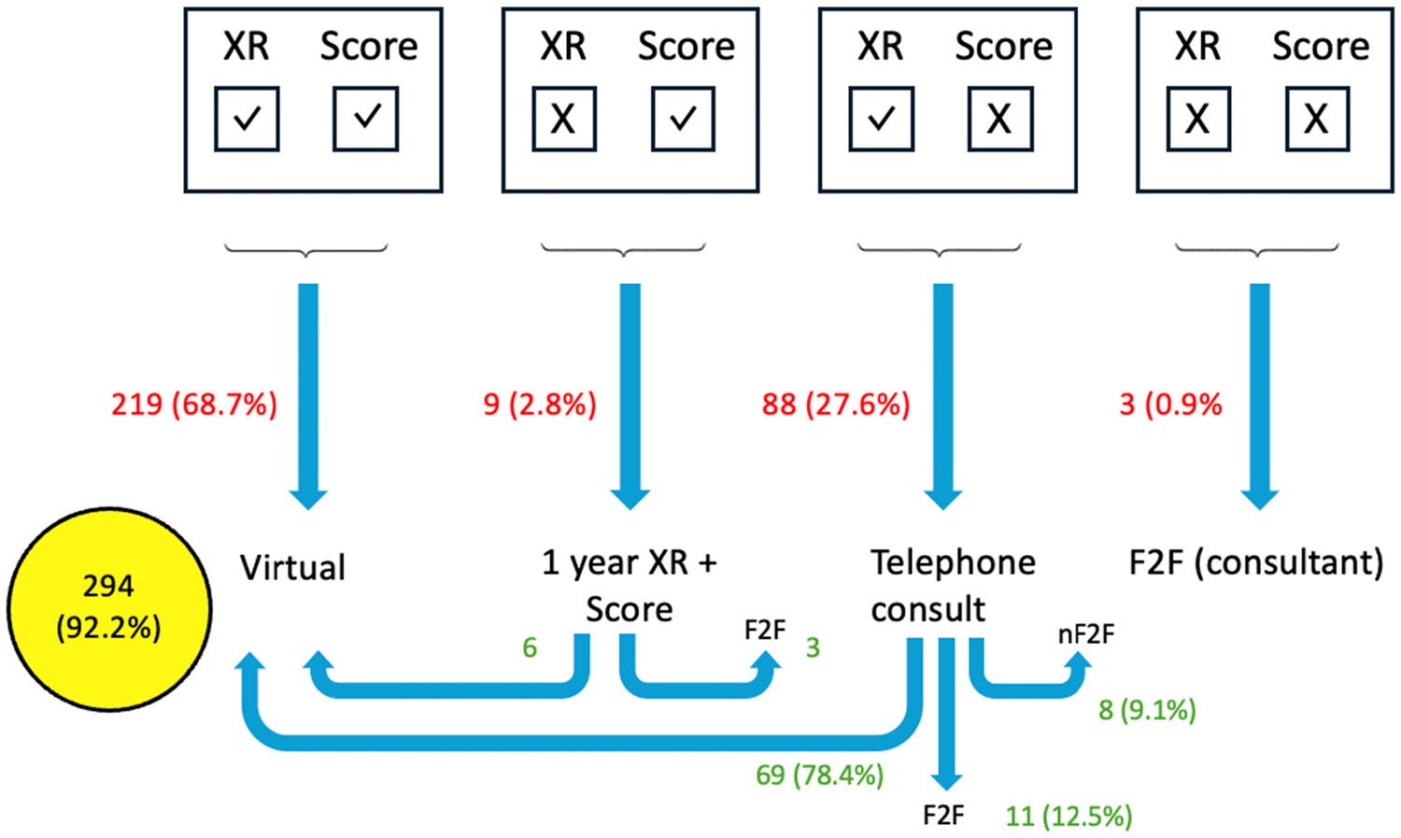


Among these, 68.7% (*n* = 219) had both satisfactory radiographs and OHS and were enrolled directly into VARF. A further 2.8% (*n* = 9) had satisfactory OHS but unsatisfactory radiographs. Following triage by the consultant, six patients were re-enrolled into VARF, while three required face-to-face review with a consultant.

In addition, 27.6% (*n* = 88) presented with satisfactory radiographs but poor OHS. After telephone assessment, 69 patients (78.4% of this subgroup) were returned to VARF. 12.5% (*n*  =  11) of this subgroup were reviewed face-to-face by a consultant and 9.1% (*n* = 3) were consulted via telephone appointment. Finally, 0.9% (*n* = 3) of patients had both unsatisfactory radiographs and OHS. All three of these patients were reviewed face-to-face by a consultant.

Overall, 92.2% (*n* = 294) of the total cohort were ultimately managed within the virtual follow-up pathway.

All patients underwent total hip replacement using a DePuy Synthes C-Stem via a posterior approach [12]. The primary operating surgeon was a consultant in 69% of cases (*n* = 221), and a registrar in the remaining 31% (*n*  = 98). A total of 68% of procedures (*n* = 216) were elective cases, while 32% (*n*  = 103) were carried out for trauma. Regarding complications, one patient required return to theatre 7 days post-operatively for washout of a large haematoma. This developed during their index admission following recommencement of regular direct oral anticoagulant (DOAC) therapy.

### Oxford Hip Scores

**Fig. 3:**
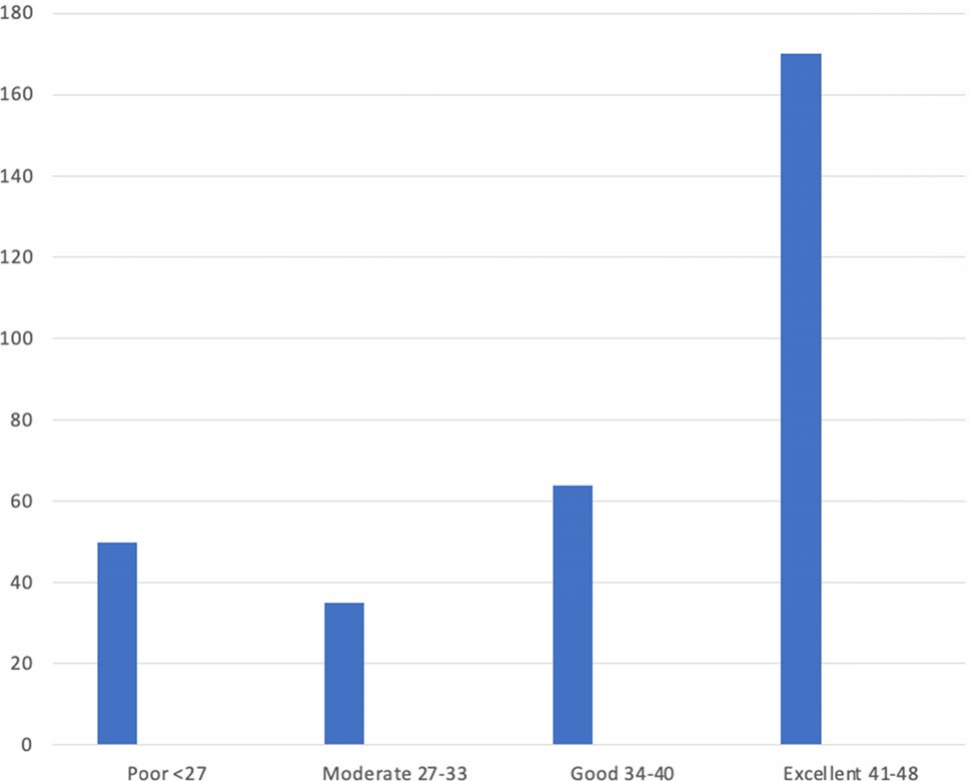
Oxford Hip Scores

As shown in Fig. 3, 53% of patients achieved ‘Excellent’ outcome scores (41–48), while 73% demonstrated either ‘Good’ (34–40) or ‘Excellent’ scores overall.

### Patient satisfaction survey

A sample of 35 patients was contacted by telephone to evaluate satisfaction with the VARF programme. Demographic characteristics of the sample are summarised below:

**Table:** 

Patients contacted	35
Mean age of patients	78.5 years
Total distance travelled	26.4 miles
Oxford Hip Score	38.4

**Fig. 4:**
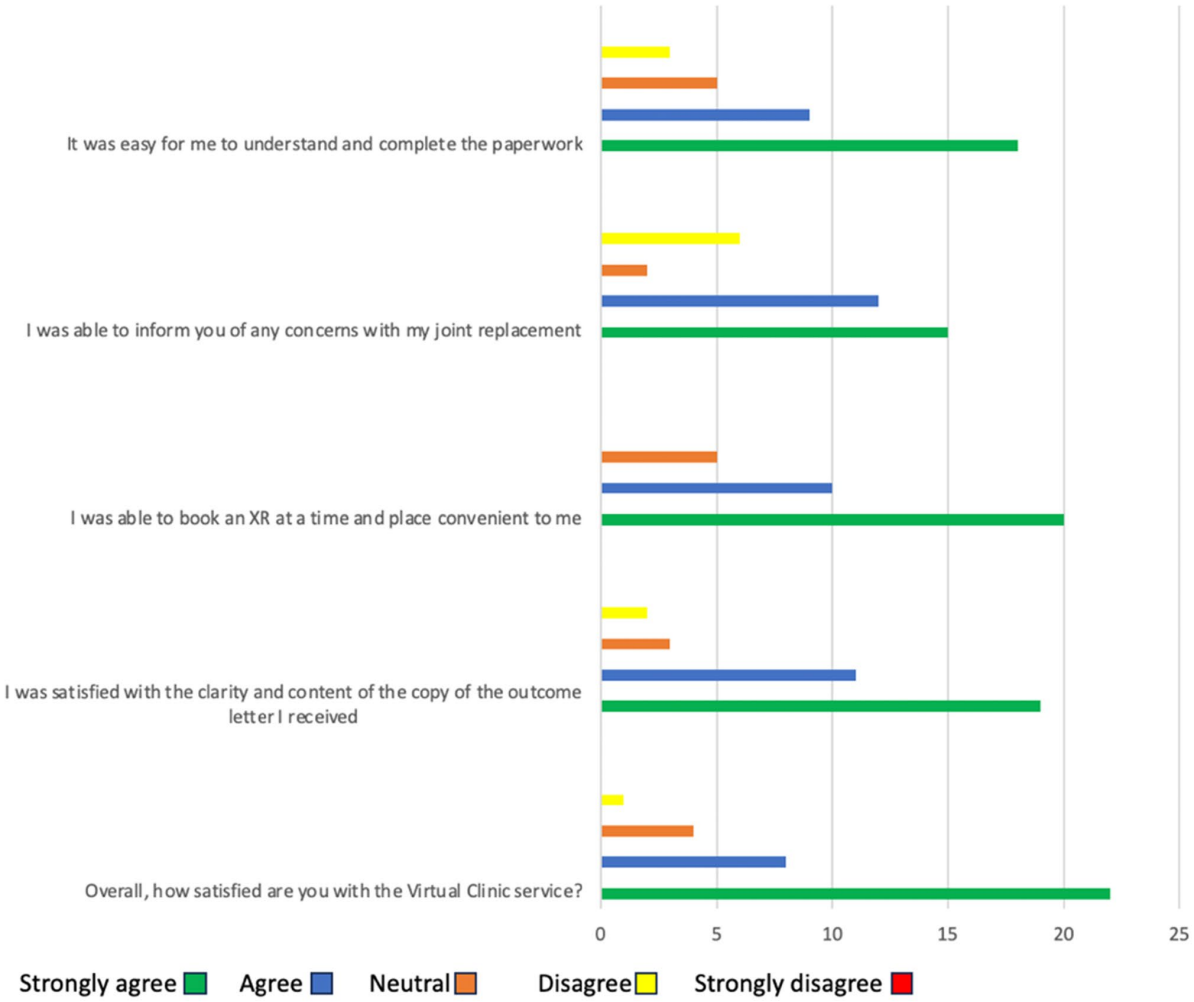
Likert-scale survey results

The results of the Likert-scale survey are shown in Fig. 4. Overall, 86% of respondents reported being at least ‘satisfied’, with 63% ‘very satisfied’. Only one patient (3%) reported being ‘dissatisfied’.

This subgroup was broadly representative of the full cohort of 319 patients, whose average age was 73 years, and mean travel distance was 32.8 miles.

Regarding perceived benefits, 69% (24 patients) felt the service saved both time and money; 9% (3) felt it saved time only; and 14% (5) felt it saved money only. Additional qualitative feedback highlighted further advantages: patients who disliked hospital environments valued avoiding in-person visits, and those without personal transport appreciated not needing to rely on friends or family for assistance.

### Financial benefit

At the time of review, the estimated cost per mile for an average UK petrol car is 17 p, based on a fuel price of £1.35 per litre [13, 14]. With a mean return trip distance of 32.8 miles and parking costs of £6.20 for two hours at Musgrove Park Hospital, the estimated average saving per patient driving a petrol car is £11.78 [15].

Additionally, face-to-face appointments are scheduled for 20 min per patient, compared with 10 min for VARF consultations. This equates to 28.8 consultant clinic lists required for in-person reviews versus 14.4 for VARF. A face-to-face clinic session costs £1922 compared to £446 for VARF. For the 302 out of 319 patients managed virtually, this translates to a potential cost-saving of £48,978.91.

While these savings are considered theoretical—given that fixed costs such as clinic space and staffing persist—the VARF model aligns with NHS priorities by releasing outpatient capacity. This enables more efficient use of existing resources, allowing face-to-face appointments to be prioritised for patients with greater clinical need, without incurring additional cost. In this way, VARF supports the NHS Long Term Plan’s goals of optimising outpatient care and reducing unnecessary hospital visits [16].

### Environmental benefit

Avoiding face-to-face follow-up appointments delivered a measurable reduction in carbon emissions. The estimated carbon footprint of a single in-person outpatient visit—including energy use for heating, lighting, and waste—is between 56 and 76 kg CO_2_e per patient, resulting in a total estimated saving of 16,912–22,952 kg CO_2_e across the cohort. Additionally, by eliminating the need for patient travel—an average of 32.8 miles per patient—a further 2779 kg CO_2_e was saved, calculated using emissions data from the Royal Automobile Club (RAC) and the Department for Energy Security and Net Zero (302 patients × 32.8 miles × 0.28052 kg CO_2_e per mile) [17, 18].

While heating and lighting are fixed infrastructural costs, they are included in NHS environmental accounting frameworks and represent the marginal emissions attributable to running outpatient services. Therefore, they remain relevant for sustainability evaluations.

In total, for the 302 out of 319 patients who were successfully managed without face-to-face review, the estimated carbon saving was approximately 19,691 kg CO_2_e**,** equivalent to 65.2 kg CO_2_e per patient (using the lower bound estimate).

## Discussion

The VARF programme has been shown to be a cost-saving, sustainable, and clinically safe approach to post-operative monitoring after THR. The high proportion of patients (92.2%) successfully managed through virtual follow-up, without compromising outcomes, strongly supports the integration of virtual care models into orthopaedic practice. The combination of OHS and radiographic assessments demonstrates that virtual programmes can effectively monitor recovery, with only a small fraction of patients requiring in-person review, thereby confirming the clinical feasibility of remote follow-up for THR.

The results align with the wider literature on virtual arthroplasty programmes [4]. Patient satisfaction in this cohort was high, with 86% reporting they were ‘satisfied’ or ‘very satisfied’ with VARF. These findings mirror those of the Richards [11] pilot study, reinforcing a consistent patient preference for virtual care [11]. While only 11% of the total cohort were surveyed—potentially limiting the representativeness of these results—the alignment with previous pilot data lends credibility to their reliability.

In our cohort of 319 patients, 84.9% achieved an Oxford Hip Score (OHS) of 34 or higher, while 15.1% fell into the poor-to-moderate range (≤ 33). These results closely mirror those reported by Devane et al. who analysed 17,831 primary total hip replacements recorded in the New Zealand Joint Registry [19]. At 6 months post-operatively, 85% of patients scored ≥ 34 and 15% scored ≤ 33. Even at 5 years, based on 3,665 OHS records, the figures remained comparable, with 89.4% scoring ≥ 34 and 10.6% scoring ≤ 33. Our outcomes are particularly notable given that 32% of our cohort underwent surgery for fracture—a proportion far higher than the 3.5% of acute fracture cases reported in the New Zealand registry—yet our overall results still align closely with elective registry data.

While traditional reporting of Oxford Hip Score (OHS) has relied on grouped scoring categories, there is a growing trend towards using outcome thresholds that may offer more patient-centred interpretations [20–22]. Harris et al. [23] proposed a Patient Acceptable Symptom State (PASS) OHS threshold of 30.6 and a Treatment Failure (TF) OHS threshold of 25.5 at 12 months post-THR. These thresholds attempt to reflect how patients themselves define success and failure: scores above PASS correspond to acceptable symptoms, while those below TF indicate dissatisfaction or failure. In our cohort, 268 patients (84.0%) met or exceeded the PASS threshold, and just 20 patients (6.3%) fell below the TF threshold. However, multiple PASS and TF thresholds have been proposed, with variability across populations, methodologies, and follow-up periods. While these thresholds may offer more meaningful context than raw banding, their application is not yet standardised. Ongoing work may help refine these cut-offs or support the development of alternative metrics better suited to tracking patient satisfaction and functional outcomes—particularly within evolving care models like virtual follow-up.

While PROM data are central to virtual follow-up, scoring systems such as the OHS have inherent limitations. In our study, 88 patients had satisfactory radiographs, but poor OHS scores. Notably, 78.4% of this subgroup were ultimately re-enrolled into VARF after further assessment. As detailed in Appendix A, several OHS questions are not specific to the operated hip. For example, questions like ‘Have you been able to climb a flight of stairs?’ or ‘Could you do the household shopping on your own?’ may reflect limitations caused by unrelated factors, such as contralateral hip or knee pain, or spinal pathology, despite patients being satisfied with their hip replacement outcomes. As a result of OHS limitations, 88 patients required additional review, with 19 ultimately needing consultant input, either via telephone (eight patients) or face-to-face (11 patients).

These limitations reflect growing recognition that the Oxford Hip Score may not fully capture what matters most to today’s patients. Holmenlund et al. called for its revision, highlighting the omission of important areas such as physical activity and overall quality of life [24]. Their findings reinforce the idea that the OHS is better suited as one component of a broader assessment, rather than the sole measure of outcome, and support the case for developing more up-to-date and patient-focussed PROM tools.

Beyond favourable PROMs, qualitative feedback revealed additional advantages of the VARF programme. Many patients described reduced anxiety related to hospital attendance—particularly those who are uncomfortable in clinical environments or face challenges with transport. This highlights the broader psychosocial value of virtual follow-up, reinforcing its role in patient-centred care.

However, it is important to acknowledge potential barriers. Virtual platforms may be less accessible to some individuals, especially older adults or those with limited digital literacy or access to technology [25–27]. Nonetheless, evidence from the COVID-19 pandemic suggests that older adults have shown a rapid adaptation to digital health care, increasingly using telehealth, smartphone applications, and other digital tools to maintain communication with providers and support self-management [28, 29].

This study has several limitations. It is a single-centre, retrospective analysis with a relatively short follow-up period. Larger, multi-centre studies with longer follow-up are warranted to validate and expand upon these findings. A total of 60 patients were censored due to non-response despite repeated invitations, relocation, or death. Consequently, we were unable to compare demographic characteristics between respondents and non-respondents, limiting our ability to assess potential selection bias and affecting the generalisability of our results—particularly regarding age or other demographic factors.

Although VARF demonstrates significant cost-savings for the Trust, it is important to acknowledge hidden costs, such as administrative tasks involved in coordinating radiographs, distributing patient questionnaires, and scheduling additional consultant review sessions to interpret results.

A potential criticism of virtual follow-up is the risk of missing post-operative complications. In our cohort, we had one patient with a large haematoma requiring washout at 7 days post op, but no additional complications were identified through the 1 year follow-up process; however, we acknowledge that some issues—particularly minor or subacute complications—may be managed in the community by general practitioners (GPs) and therefore not be captured in our system.

Nevertheless, the most clinically significant complications following THR, such as periprosthetic joint infection (PJI) or periprosthetic fracture, typically present acutely and prompt emergency hospital attendance. As a result, these would still be detected regardless of follow-up format. To mitigate this risk, all patients attend a pre-operative joint school where they are clearly advised on potential warning signs and the importance of seeking timely medical attention. By combining robust patient education with ongoing access to emergency care, this model remains a safe and pragmatic alternative to routine face-to-face review for the majority of uncomplicated THR patients.

Another consideration is the potential reduction in opportunities for operating surgeons to receive direct, nuanced feedback from patients. Traditional clinic reviews allow for physical examination and personal interaction, which may be lost in a virtual setting. To address this, structured feedback mechanisms led by Clinical Nurse Practitioners—encompassing red-flag escalation and documentation of functional progress—can serve as a proxy for surgeon engagement, helping to maintain clinical oversight and support ongoing quality improvement within the virtual follow-up pathway.

Overall, VARF represents an effective, patient-centred, and environmentally conscious model for post-THR care. However, careful consideration of its limitations and targeted refinements to PROMs and feedback processes will be essential for its long-term success and broader adoption.

## Conclusion

The implementation of the virtual arthroplasty follow-up (VARF) programme for total hip replacement patients has been shown to be clinically effective, resource-efficient, and highly acceptable to patients. The vast majority of patients were safely managed remotely, demonstrating excellent functional outcomes, high satisfaction levels, significant cost-savings, and notable environmental benefits.

By reducing the need for in-person clinic visits, VARF alleviates logistical and financial burdens on patients while optimising outpatient capacity for those requiring face-to-face review. This approach aligns with broader healthcare priorities of delivering patient-centred, sustainable, and value-based care.

As healthcare systems continue to seek innovative strategies to improve efficiency and patient experience, VARF stands out as a scalable model for post-operative arthroplasty care. Future work should focus on expanding this framework to include total knee replacement (TKR) patients, to further establish the role of virtual follow-up across different joint replacement populations.

## Supplementary Information

Below is the link to the electronic supplementary material.

Supplementary file1 (DOCX 14 kb)

## Data Availability

No datasets were generated or analysed during the current study.
