# Supplementary material for: Virtual arthroplasty follow-up (VARF) in total hip replacement: a safe, effective, and sustainable model for post-operative care
Source: Eur J Orthop Surg Traumatol. 2025 Aug 26;35(1):361. doi: 10.1007/s00590-025-04479-y (PMC12380640; doi:10.1007/s00590-025-04479-y)
Supplement: Supplementary file 1 — Supplementary file1 (DOCX 14 kb) [file 590_2025_4479_MOESM1_ESM.docx]

**Appendix A**

Patient Satisfaction Questionnaire VARF

(1) It was easy for me to understand and complete the paperwork:

Strongly agree      Agree      Neither agree nor disagree      Disagree Strongly      Disagree

(2) I was able to inform you of any concerns with my joint replacement(s) (this may also include via a requested telephone call):

Strongly agree      Agree      Neither agree nor disagree      Disagree Strongly      Disagree

(3) I was able to book the X-ray at a time and place convenient to me:

Strongly agree      Agree      Neither agree nor disagree      Disagree Strongly      Disagree

(4) I was satisfied with the clarity and content of the copy of the outcome letter I received following Virtual Clinic review:

Strongly agree      Agree      Neither agree nor disagree      Disagree Strongly      Disagree

(5) Did you also receive a telephone call to further discuss the joint replacement?

Yes, please continue below; No, please move to Q6

I was satisfied with the outcome of the telephone call:

Strongly agree      Agree      Neither agree nor disagree      Disagree Strongly      Disagree

(6) Did you also attend an outpatient appointment following on from, or instead of, Virtual Clinic review?

Yes, please continue below; No, please move to Q7

I was satisfied with the outcome at the Orthopedic Outpatient Appointment following further assessment of the joint replacements(s):

Strongly agree      Agree      Neither agree nor disagree      Disagree Strongly      Disagree

The appointment was with:

An orthopedic surgeon      A specialist physiotherapist

(7) Do you Feel the Virtual Clinic saved you any time or money compared to a standard outpatient appointment?

Time      Money      Both      Neither

If yes, please tick all reasons that apply:

Travel distance      Travel time      Travel costs      Parking costs      Time off work Less wait time in clinic

Other (please give details)…………………………………………………

(8) Overall, how satisfied are you with the Virtual Clinic service?

Very satisfied      Satisfied      Neither satisfied nor dissatisfied      Dissatisfied      Very dissatisfied

(9) If you have any comments on how we could improve the service, please tell us (please continue on the back page if you like)

10) What would be your preferred method of future routine joint replacement review (normally 1, 7, and 10 years following surgery, then every 3 years)

- Virtual Clinic (as the previous review, with option of formal appointment if concerns)

- Web-based follow-up (email and electronic questionnaires to complete on a computer, tablet, or suitable phone, with the option of formal appointment if concerns)

- Formal outpatient clinic appointment at Musgrove Park Hospital

- None, I would rather be discharged to the care of my GP
